# Race Against the Clock: Severe Acute Abdominal Pain With Disastrous Complications

**DOI:** 10.7759/cureus.76082

**Published:** 2024-12-20

**Authors:** Tushita S Verma, Charles Barrows, Patricia E Souchon Sanchez, Anna L Koenig, Sara Assaf

**Affiliations:** 1 Internal Medicine, University of New Mexico Hospitals, Albuquerque, USA; 2 General Surgery, University of New Mexico Hospitals, Albuquerque, USA; 3 Pulmonary, Critical Care and Sleep Medicine, University of New Mexico Hospitals, Albuquerque, USA

**Keywords:** clinical case report, critical care and hospital medicine, gastric necrosis, septic shock, undifferentiated shock

## Abstract

Although a rare medical condition, gastric ischemia is a medical emergency and requires prompt recognition. Current literature review is scarce, with a primary focus on iatrogenic, surgical, and vascular etiologies. The cases discussed focus on hypoperfusion secondary to refractory shock as the etiology of gastric ischemia and, unfortunately, death. Our cases add to the current literature by highlighting an alternative, less common etiology, thus broadening a clinician’s suspicion of gastric ischemia complications in critically ill patients.

## Introduction

Gastric ischemia is a rare medical emergency due to the vast vascular supply of the stomach [1]. Presenting symptoms are non-specific and range from vague abdominal pain and nausea to weight loss and vomiting. Diagnostic evaluation includes computed tomography (CT), CT angiography (CTA), and endoscopy to determine patency of vasculature and the full extent of gastric ischemia [2]. The mainstays of treatment include vascular support, antibiotics, proton-pump inhibitor (PPI) therapy, and nasogastric suction if gastric distension is present, with a focus on the inciting cause of ischemia, hoping that appropriate treatment may decrease mortality [2]. 

A typical management approach is to first differentiate between acute versus chronic gastric ischemia. The former is often caused by acute insults such as hypotension, sepsis, and acute trauma and can be thought of as something that is possibly reversible if blood flow is improved [2]. The latter can be attributed to structural abnormalities, obstructive pathologies, or gastric volvulus [2]. Gastric ischemia is frequently missed due to its non-specific symptoms that coincide with more prominent medical conditions such as peptic ulcer disease or gastritis [3]. Prior case reports have discussed gastric ischemia as an unfortunate complication of hypoperfusion/vascular complications, abdominal distension, and gastroenterological intervention [1-10]. 

Limited information is available on gastric ischemia secondary to refractory shock requiring the use of high-dose vasopressors. The cases below demonstrate patients with refractory shock secondary to perforated peptic ulcer disease and unknown etiology. Case one regarding gastric necrosis secondary to isolated refractory shock was previously presented as a meeting abstract at the 2023 CHEST Conference on October 11th, 2023. 

## Case presentation

Case 1

A 65-year-old female, a long-term smoker with chronic obstructive pulmonary disease, obstructive sleep apnea not on continuous positive airway pressure (CPAP), hypertension, and atrial fibrillation not on anticoagulation, presented to the emergency department (ED) with five days of worsening shortness of breath, severe abdominal pain, obstipation, and non-bloody bilious emesis. On initial evaluation, the patient was afebrile with a heart rate of 135 bpm, hypotensive (BP 80/50, mean arterial pressure (MAP) 50s), and had increased work of breathing, saturating 83% on room air. Subsequently, non-invasive positive pressure ventilation was initiated. The physical exam was concerning for the acute abdomen as the patient had peritoneal signs, a distended abdomen, and absent bowel sounds. Laboratory testing and patients’ prior medications are seen in Tables 1, 2.

**Table 1:** Medication list for patient 1.

Medication Name	Route of Administration
Albuterol HFA inhaler	As needed every 4 hours for shortness of breath
Hydrochlorothiazide (HCTZ) – triamterene 50-75mg	By mouth once daily
Metoprolol tartrate 50mg	By mouth once daily

**Table 2:** Lab values on initial presentation for patient 1. ALT: alanine aminotransferase; AST: aspartate aminotransferase; WBC: white blood cells

Laboratory Testing	Patient’s Initial Lab Values	Reference Range/Normal
WBC	10.7x10^9^/L	4.0–11.0 X 10E3/UL
Creatinine	3.41 mg/dL	0.7-1.35 mg/dL
AST	5207 U/L	6-58 U/L
ALT	2369 U/L	14-67 U/L
Alkaline phosphatase	152 U/L	38 – 150 U/L
Anion gap	23	<15
Lactate	13 mmol/L	< 2 mmol/L

**Figure 1:**
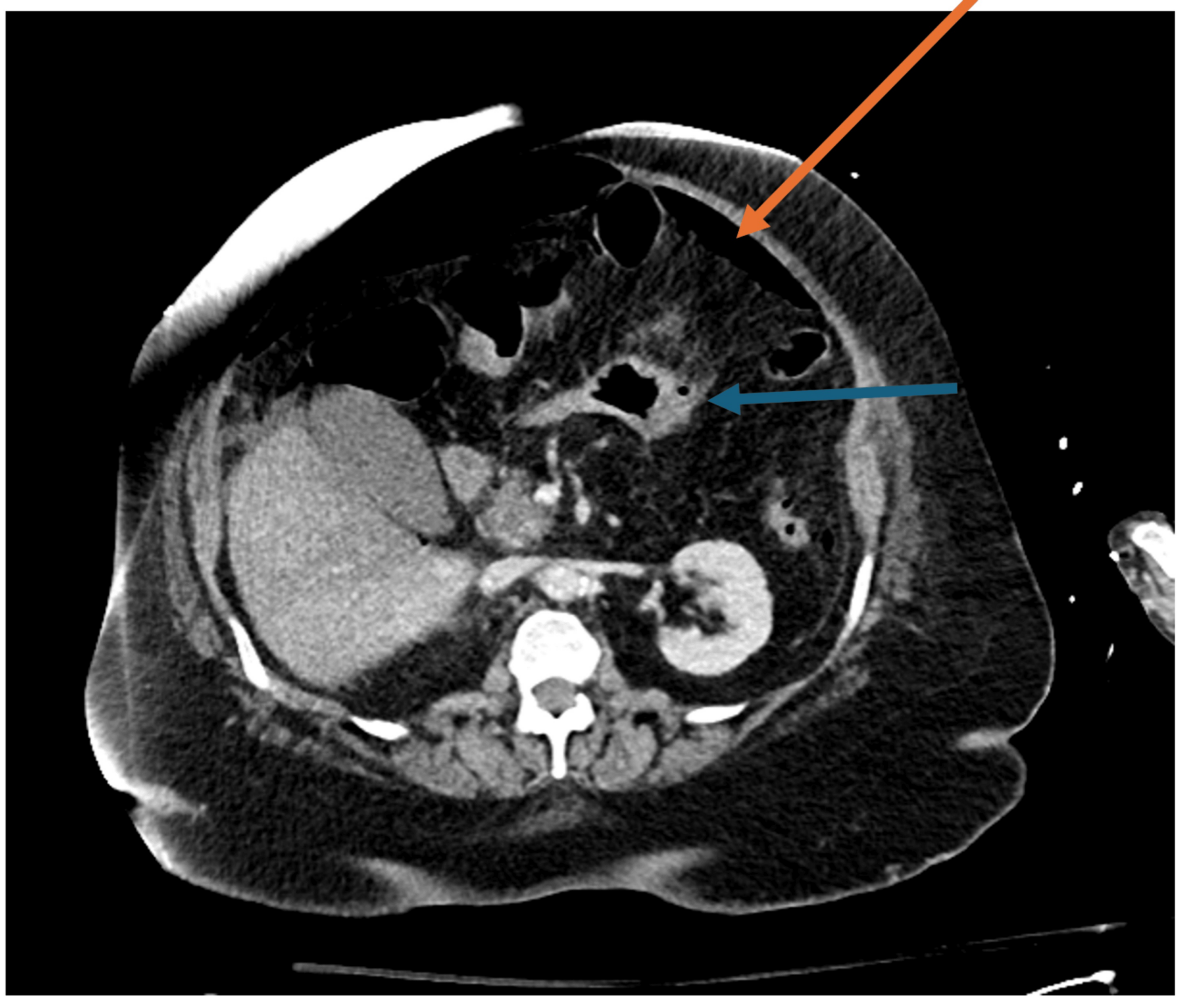
Computed tomography demonstrating gastric thickening with massive pneumoperitoneum outside of gastric wall. Orange arrows point to the pneumoperitoneum. Blue arrows point to gastric thickening.

**Figure 2:**
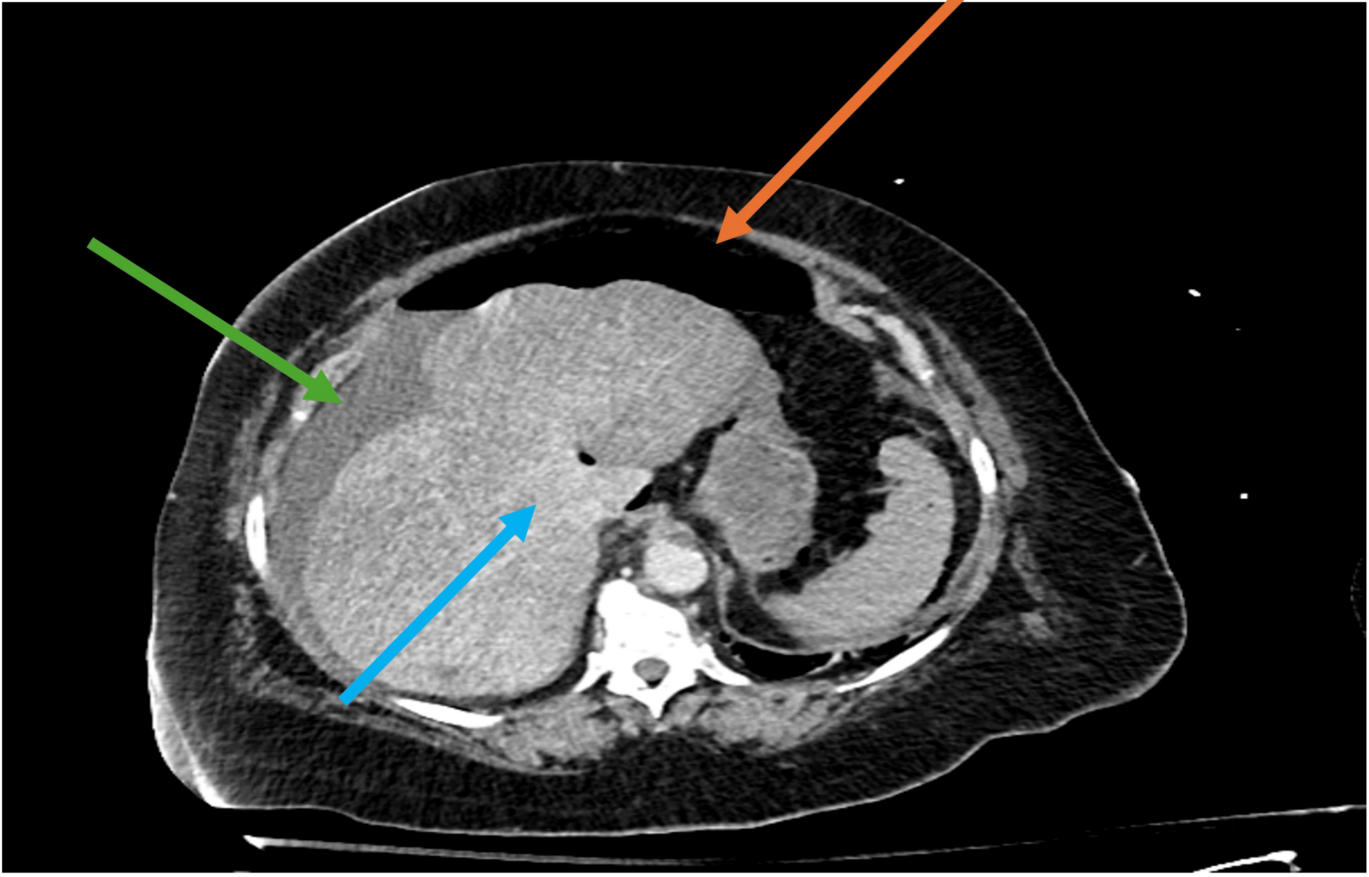
Computed tomography demonstrating liver hypo attenuation. Orange arrow points to pneumoperitoneum. Green arrow points to free fluid. Blue arrow highlights area of liver hypo-attenuation.

Computed tomography (CT) abdomen/pelvis with intravenous (IV) contrast showed mural thickening along the stomach's greater curvature and a large pneumoperitoneum (Figures 1, 2). Surgery was consulted, and the patient was started on empiric antibiotic therapy with ceftriaxone, metronidazole, and micafungin. Despite fluid resuscitation, she had progressively worsening shock requiring multiple pressors, and was intubated. She was then taken emergently to the operating room (OR). She underwent an exploratory laparotomy, abdominal washout, and modified Graham patch placement for peptic/gastric ulcer. A temporary closure was performed given the patient's hemodynamic instability, and she was transferred to the intensive care unit (ICU), continuing to require three vasopressors. Her vasopressor use peaked on day one of hospitalization at norepinephrine at 30 mcg/min, phenylephrine at 300 mcg/min, and vasopressin at 0.08 mcg/min. She then developed anuric renal failure and was initiated on renal replacement therapy. Two days later, her lactic acidemia continued to worsen, and she was taken back to the OR with findings of total gastric ischemia and dehiscence of her previously repaired gastric ulcer in addition to a new perforation. In total, 50 cm of ischemic bowel and ischemic liver were appreciated, all attributed to her refractory septic shock. Given her multiorgan failure, an intraoperative discussion with the patient's family was facilitated. Given the severity of her condition, the decision was made to refrain from any further organ resection, and instead, a transition was made to focus on comfort measures. The patient expired later that day.  

Case 2

**Figure 3:**
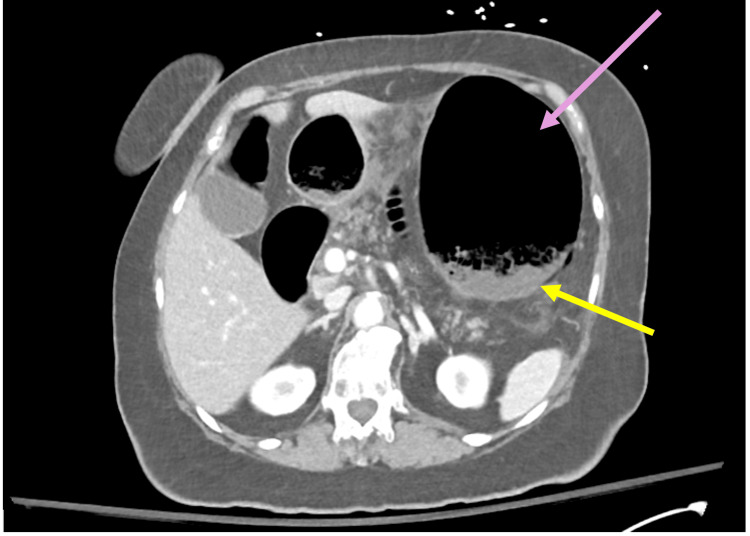
Computed tomography demonstrating gastric pneumatosis and stomach dilation. Purple arrow points to gastric dilation. Yellow arrow points to pneumatosis.

A 73-year-old female with hypothyroidism, hypertension, acid reflux, and type 2 diabetes mellitus was nonadherent with her medications one week prior to arrival secondary to feeling ill, and presented to the ED with acute abdominal pain for one week (Table 3). On initial evaluation, the patient had a respiratory rate of 36 bpm, tachycardia of 101 bpm, and BP of 198/102. The physical exam was pertinent for increased work of breathing and a distended, firm abdomen with tenderness to palpation without peritoneal signs. Relevant laboratory testing is seen in Table 4. Admission CT abdomen/pelvis with IV contrast demonstrated pneumatosis, duodenitis with concern for ulcer, and concern for low-grade bowel obstruction (Figure 3). 

**Table 3:** Medication list for patient 2.

Medication Name	Route of Administration
Glipizide 5mg	By mouth once daily
Pantoprazole 40mg	By mouth once daily
Losartan 25mg	By mouth once daily
Hydrochlorothiazide (HCTZ) 25mg	By mouth once daily
Fenofibrate 150mg	By mouth once daily
Levothyroxine 100mcg	By mouth once daily

**Table 4:** Lab values on admission for patient 2 ALT: alanine aminotransferase; AST: aspartate aminotransferase; WBC: white blood cells; BUN: blood urea nitrogen

Laboratory Testing	Patient’s Initial Lab Values	Reference Range/Normal
WBC	22.4 x10^9^/L with neutrophilia	4.0–11.0 X 10E3/UL
BUN	34 mg/dL	7-25 mg/dL
Creatinine	1.07 mg/dL	0.7-1.35 mg/dL
Glucose, serum	536 mg/dL	60–100 mg/dL
Anion gap	19	< 15
ALT	41 units/L	10-50 units/L
AST	37 units/L	10-50 units/L
Lipase	28,472	13–60 U/L
Lactate	5.5 mmol/L	<2 mmol/L

She was admitted to the ICU, where an attempt to decompress the abdomen with nasogastric (NG) tube placement was unsuccessful. This patient started antimicrobial therapy with piperacillin-tazobactam and micafungin, along with intravenous fluids. Within 24 hours of presentation, she became more unstable with worsening abdominal distension and pain. A bedside kidney, ureter and bladder (KUB) was concerning for pneumoperitoneum. Surgery was reconsulted, and she was then intubated and taken for an emergent exploratory laparotomy. Intraoperatively, she progressively got more hypotensive, requiring initiation of norepinephrine, vasopressin, and phenylephrine (peak rate of 55 mcg/min, multiple push doses of 0.1 mL IV injections, and multiple push doses of 20 mcg IV, respectively). She was found to have gross gastric necrosis up until her hiatal hernia and perforation on the greater curvature of her stomach without optimal surgical remedies. With increasing pressor requirements and hemodynamic instability during surgery, a mid-operation discussion with the family was facilitated. Due to the severity of ischemia and the likely futility of further surgical intervention, the family decided to transition the goals of treatment to focus on comfort. The patient expired later that day. 

## Discussion

Case analysis and differential diagnosis

The cases above demonstrate the rapid nature of gastric ischemia and necrosis, and our differential diagnosis was limited. In case one, a CT scan of the abdomen with IV contrast revealed patent abdominal vasculature. It was thought that refractory shock requiring high-dose pressors with persistent systemic hypotension could have acutely contributed to the patient’s demise. In case two, given the patient’s rapid demise, the exact etiology of her gastric ischemia was not determined. However, it can be speculated that it is secondary to chronic co-morbid conditions and the severity of shock upon her delayed presentation. No prior gastroenterological interventions or known vascular blood supply issues were noted in the chart review, making vascular etiology less likely. Despite evidence of gastric perforation, it was thought that this was secondary to patients’ comorbidities and acute shock instead of the primary source of decompensation. Chart review did not reveal any prior history of ulcer disease or intervention that could explain the perforation. 

Etiology of gastric necrosis

In both cases, gastric ischemia secondary to significant shock requiring vasopressor use is thought to be the primary cause. One theory is that the supply-demand ratio of blood supply in septic states is altered and can contribute to gastric ischemia that is then further exacerbated by altered regional blood flow secondary to the use of vasopressors [4]. Although this can usually be seen in the small and large intestines due to watershed areas and lack of collateral blood flow, it can be assumed that gastric circulation can also succumb to this in the setting of preexisting damage or blood flow limitations, despite being a rare occurrence. In one case report, it was thought that the microvascular changes induced by sepsis (i.e., increased capillary permeability) increased tissue hypoxia despite conventional resuscitation means [4]. An alternative pathophysiological explanation is the loss of gut barrier, increased translocation of gut bacteria, and endotoxin production are thought to contribute to mucosal hypoperfusion leading to ischemia [5]. Disruptions in the nitric oxide system and other vasoactive enteric mediators also can be contributors [5]. Given the scarcity of literature reviews addressing gastric ischemia secondary to shock, the above theories from prior case reports add to the speculation of the pathophysiology leading to ischemia.

Clinical management: symptoms, diagnostic workup, treatment 

Typically, symptoms are non-specific for gastric ischemia, including non-specific abdominal pain, nausea, and vomiting, mimicking a large differential of etiologies leading to late recognition [4]. In addition, laboratory findings can also be non-specific (elevated lactate and leukocytosis being common) [4]. Workup is based on clinical suspicion and should be directed at symptoms. Despite the rarity of gastric ischemia, it should remain on the physician’s differential in the absence of alternative etiologies. CT abdomen/pelvis with IV contrast can be obtained to assist with diagnosis in the appropriate clinical setting. 

Esophagogastroduodenoscopy (EGD) can be utilized for early diagnosis, severity, and extent of gastric ischemia; however, its use can be limited secondary to patient hemodynamic status [6]. On EGD, endoscopic findings can include diffuse or patchy gastric mucosa discoloration/paleness, loss of vascular pattern, erosions, or ulcerations [7]. In both cases described above, the patients were hemodynamically unstable; therefore, CT and KUB findings, respectively, prompted emergent laparotomy evaluation. Other case studies have been able to utilize EGD to determine the extent of ischemia to direct earlier management (Table 5). Even though EGD does offer valuable information, CT and CT angiography can also provide rapid evaluation in clinically unstable patients. 

**Table 5:** Summation of prior literature review and cases. ABX: antibiotics; CKD: chronic kidney disease; CT: computed tomography; EGD: esophagogastroduodenoscopy; GI bleed: gastrointestinal bleed; HTN: hypertension; PNA: pneumonia; PPI: proton pump inhibitor; SBO: small bowel obstruction; T2DM: type 2 diabetes mellitus; w/: with; Yr: year; ABPA: allergic bronchopulmonary aspergillosis

Authors	Hospital Course	Diagnostic Workup	Management/Supportive +/- Pressor Use	Etiology of Gastric Ischemia	Outcome
Thomas M (Anaesth Intensive Care) 2003 [4]	67-yr-old male with severe asthma, ABPA, and bronchiectasis. Diagnosis: Gastric infarction	CT scan showed dilated stomach with gas in stomach wall, portal system and liver and partial SBO at jejunal level	Used norepinephrine and vasopressin during ICU admission (highest rate of 35 micrograms/min and 0.02 U.min, respectively).	Severe sepsis and high dose pressor use.	Deceased
Tang et al. (Clinical Gastroenterology and Hepatology) 2014 [6]	Case series: 32-yr-old male with HTN. Diagnosis: Acute alcoholic hepatitis and septic shock; GI bleeding.	EGD showed ulceration from gastric fundus to antrum along greater curvature.	On 2 pressors, doses not listed.	Not specified.	Deceased from multiorgan failure
Chiang et al. (BMC Gastroenterol) 2021 [7]	82-yr-old male with Parkinsonism. Diagnosis: Septic shock	CT showed venous air in portal veins, thickened gastric fundus wall with gastric pneumatosis on imaging. EGD showed fundal erythema and edema and diffuse shallow fundal ulceration and hemorrhage.	No pressors.	Hypotension attributed to sepsis. The authors attributed the source of sepsis to gastric ischemia.	Improved
Contreras Saiz et al. (Cirugía Española) 2020 [9]	61-yr-old female w/ lobular carcinoma s/p mastectomy 3 years prior Diagnosis: Septic shock and gastric ischemia	Initial CT on admission showed free intra-abdominal fluid and mucosal edema in the gastric antrum and body, concerning for ischemia. Gastroscopy showed ischemic mucosa in the cardia.	Required high pressor doses (dosage not provided); refractory shock. No listed abx or PPI; underwent total gastrectomy.	Idiopathic.	Deceased
Lee et al. (Life) 2024 [10]	65-yr-old male with CKD and T2DM Diagnosis: COVID PNA, GI bleed, renal failure,	CT abdomen showed “minimal gastric perforation”. EGD was deferred. Emergent laparoscopic surgery that showed a “blue” stomach indicative of low blood perfusion (gastric ischemia) but did NOT show any acute perforation, or necrosis.	COVID treatment with remdesivir, methyl prednisone, and moxifloxacin was given. No vasopressors were documented to be used.	Not clearly identified however thought secondary to comorbid conditions that were superimposed with COVID-19 infection.	Deceased

In general, treatment includes gastric decompression, fluid resuscitation, broad-spectrum antibiotic coverage, and surgical evaluation. Revascularization is considered in cases of localized vascular insufficiency. Although gastrectomy with esophagojejunostomy anastomosis may be a possible corrective surgery, the surgery itself has a high morbidity and mortality rate in the emergency setting. Patient factors include stability, comorbidities, and goals of care that should be addressed in a risk versus benefit discussion with family members. Even in individuals with prompt recognition, gastric ischemia has a high mortality rate, around 24% within six months [6].

Potential preventive measures

Critically ill patients provide a challenge to clinicians for early identification of gastric necrosis. Initial symptoms of progressive and rapid hypotension without an obvious source, uptrending lactate, acute anuria, and vague abdominal pain/tenderness on palpation can heighten suspicion for further imaging and workup for gastric necrosis. In patients with no prior gastrointestinal concerns, the above changes should further heighten suspicion for acute gastric complications secondary to acute medical problems. 

In general, optimizing blood pressure control and limiting vasopressor use, proactive management of acute vague changes in clinical status, and early imaging can help with early identification, possibly leading to decreased mortality. 

Literature review

Literature review on gastric necrosis is scarce, with underlying etiologies ranging from congenital disorders (i.e., Ehlers’s Danlos), surgical complications (acute gastric dilation, trauma, volvulus, etc.), to vascular (i.e., thrombosis and preexisting anatomical differences) and iatrogenic (i.e., surgery, embolization, etc.) [4]. Table 5 highlights other cases found in the literature that have highlighted profound hypoperfusion as the etiology of gastric ischemia.

A retrospective review of 15 isolated gastric ischemia cases had 23% of patients with systemic hypoperfusion as the etiology for their gastric ischemia, and it was noted that approximately 53% of the patients reviewed had a history of smoking [8]. Although this is a limited analysis of cases, it leads us to believe that hypoperfusion may lead to a greater number of gastric ischemia cases and is likely underreported. Our literature review focused on shock as an etiology of gastric necrosis and documented vasopressor or lack of use. A literature review was conducted primarily through PubMed with a focus on case reports of gastric necrosis due to shock as the primary etiology. Of the found cases, five case reports were found to have shock as the primary etiology of gastric necrosis. Although this is not a complete systematic review of all the cases present in the literature, the focus of the table is to compare other case reports with documented shock as the etiology for decompensation in efforts to strengthen the limited two cases above. Despite the limited literature review included, the table summarizes a variety of case presentations that lead to gastric ischemia. The reports show that the patients who received high-dose vasopressors did have poorer outcomes [4,6,7,9,10]. The case reports are limited in their ability to demonstrate association and causation, and larger studies are needed to provide a stronger association between vasopressor and gastric ischemia. Our cases above do add to the scarcity of data prevalent, enhancing the association with hypotension/vasopressor use and gastric ischemia. 

Despite adequate resuscitation efforts (outlined by MAP >65, decreasing lactate levels), appropriate gastrointestinal perfusion may not be achieved [5]. Prior studies have shown that despite appropriate gut perfusion (identified through gut tonometry), mortality outcomes were not altered [5]. It is not within the scope of this case report to delve further into alternative methods to monitor adequate gut perfusion; however, further studies should be aimed at identifying mortality outcomes in septic patients. 

## Conclusions

Despite being a rare diagnosis, physicians should have a high suspicion of gastric ischemia in critically ill individuals. Etiologies are vast, and this condition should be on the differential when non-specific changes in clinical status are occurring in the critically ill. Even if gastric ischemia is not appreciated on presentation, critically ill patients and those requiring vasopressor therapies should always be monitored for this complication. Early identification is crucial for prompt interventions, especially if a reversible etiology is identified. Aggressive management of blood pressure, prompt imaging, and early antibiotic or possible surgical evaluation can help identify and manage gastric ischemia. Despite this, mortality rates are high, and timely intervention may still be limited in its ability to prevent mortality due to the severity of this condition.
